# Myasthenia Gravis

**DOI:** 10.4061/2011/697575

**Published:** 2012-01-24

**Authors:** Johan A. Aarli, Nils Erik Gilhus, Robert P. Lisak, Renato Mantegazza, Shigeaki Suzuki

**Affiliations:** ^1^Department of Neurology, University of Bergen, Bergen, Norway; ^2^Department of Clinical Medicine, University of Bergen, Bergen, Norway; ^3^Division of Neuroimmunology, Department of Neurology, Wayne State University, Detroit, MI 48202, USA; ^4^Department of Neurology IV, Neuromuscular Diseases and Neuroimmunology, Fondazione Istituto Neurologico “Carlo Besta”, Milan, Italy; ^5^Department of Neurology, Keio University School of Medicine, Tokyo, Japan

Myasthenia gravis (MG) is the best defined autoimmune disturbance with the production of antibodies to the *n*-acetylcholine receptor (AChR) as dominating feature. Some MG patients, who do not have AChR antibodies, have antibodies to muscle-specific kinase (MUSK). In addition, some MG patients, especially those with a tumour of the thymus gland, have antibodies to other muscle proteins such as titin, ryanodine receptor (RyR), and voltage-gated potassium receptor. The role of virus infection of thymic cells and of cytokines and CD4+ cells in the pathogenesis of MG is a potential clue to the disease mechanisms in MG.

 The main focus of this special issue is the characterization of the autoimmune responses in MG, the relationship between the immunological disturbances and the clinical picture, the role of the thymus, and the specific problems related to paediatrics and anaestiology. The special issue will summarize the most recent developments in the area.

Considerable data support the involvement of thymus in the aetiology of MG. P. Cavalcante et al. address the question of whether inflammation and Epstein-Barr virus (EBV) infection are frequent pathogenic features of MG thymus. By using Low-Density Array and real-time PCR, the authors show that the MG thymic transcriptome is characterized by upregulation of genes implicated in inflammation and immune response, delineating a peculiar inflammatory and antiviral signature. By using more sensitive molecular and immohistochemical techniques, the authors have implemented and improved their previous finding of an active EBV infection in MG thymus. Hence, a new pathogenic model of virus-mediated autoimmunity in MG is proposed. EBV infection may contribute to MG-specific autoimmune responses occurring within a chronically inflamed MG thymus, through its ability to promote activation, survival, and expansion of autoreactive B cells. The overall results of the study strongly suggest that inflammation and EBV infection are key events in the intrathymic pathogenesis of MG.

In the subsequent paper, S. Ragheb and R. P. Lisak discuss the role of the B-cell activating factor (BAFF) in the pathogenesis of MG. Three independent studies have shown higher BAFF levels in the circulation of MG patients. BAFF is a potent B-cell survival factor, and it plays an essential role in B-cell homeostasis and B-cell function in the periphery.

Both normal and autoreactive B cells are BAFF dependent; however, excess BAFF promotes the survival, growth, and maturation of autoreactive B cells. When over-expressed, BAFF protects B cells from apoptosis, thereby contributing to autoimmunity. BAFF may provide new treatment options for MG patients, particularly those patients with thymic lymphoid follicular hyperplasia.

One-half of patients with cortical thymoma develop MG, while 15% of MG patients have thymomas. F. Romi has evaluated the diagnosis and treatment of MG in patients with thymoma. Titin and RyR antibodies are found in 95% of thymoma MG, and 50% of late-onset MG (MG-onset > 50 years) are associated with severe disease and may predict thymoma MG outcome. Nonlimb symptom profile at MG onset with bulbar-, ocular-, neck-, and respiratory symptoms should raise the suspicion about the presence of thymoma in MG. The presence of titin and RyR antibodies in an MG patient younger than 60 years strongly suggests a thymoma, while their absence at any age strongly excludes thymoma. Thymoma should be removed surgically. Pre-thymectomy plasmapheresis/iv-IgG should be considered before thymectomy. The pharmacological treatment does not differ from nonthymoma MG, except for tacrolimus which is an option in difficult thymoma and nonthymoma MG cases with RyR antibodies.

Patients with autoimmune MG should be further classified before initiating therapy, as treatment response varies for ocular versus generalised, early onset versus late onset, and acetylcholine receptor antibody positive versus MuSK antibody positive disease. G. O. Skeie and coworkers discuss available treatment approaches in MG. Most patients need immunosuppression in addition to symptomatic therapy. Prednisolone and azathioprine represent first-choice drugs, whereas several second-choice options are recommended and should be considered. Thymectomy should be undertaken in MG with thymoma and in generalised, early-onset MG. For MG crises and other acute exacerbations, intravenous immunoglobulin (IvIg) and plasma exchange are equally effective and safe treatments. Children and females in childbearing age need special attention regarding potential side effects of immunosuppressive therapy. MG pathogenesis is known in detail, but the immune therapy is still surprisingly unspecific, without a pinpointed attack on the defined disease-inducing antigen-antibody reaction being available.

Treatment of juvenile MG raises special problems which are discussed by J. Jayawant and N. Finnis. Juvenile MG has many clinical features that are distinct from adult MG. Pre-pubertal children in particular have a higher prevalence of isolated ocular symptoms, lower frequency of acetylcholine receptor antibodies, and a higher probability of achieving remission. Diagnosis in young children can be complicated by the need to differentiate from congenital myasthenic syndromes, which do not have an autoimmune basis. Treatment commonly includes anticholinesterases, corticosteroids with or without steroid-sparing agents, and newer immune modulating agents. Plasma exchange and intravenous immunoglobulin (IVIG) are effective in preparation for surgery and in treatment of myasthenic crisis. Thymectomy increases remission rates. Diagnosis and management of children with juvenile MG should take account of their developmental needs, natural history of the condition, and side-effect profiles of treatment options.

The autoimmune response in MG is heterogeous, but may provide more specific and useful diagnostic information, as discussed by S. Suzuki et al. MG is caused by antibodies that react mainly with the acetylcholine receptor on the postsynaptic site of the neuromuscular junction. A wide range of clinical presentations and associated features allow MG to be classified into subtypes based on autoantibody status. Striational antibodies, which react with epitopes on the muscle proteins titin, ryanodine receptor (RyR), and Kv1.4, are frequently found in MG patients with late onset and thymoma. Anti-titin and anti-RyR antibodies are determined by enzyme-linked immunosorbent assay or immunoblot. More recently, a method for the detection of anti-Kv1.4 autoantibodies has become available, involving 12–15% of all MG patients. The presence of striational antibodies is associated with more severe disease in all MG subgroups. Anti-Kv1.4 antibody is a useful marker for the potential development of lethal autoimmune myocarditis and response to calcineurin inhibitors.

Some disease mechanisms which may occur in MG, such as the presence of matrix metalloproteinase-3 (MMP-3), may also have a specific pathogenetic effect. High MMP-3 levels in a proportion of MG patients have been reported. This is discussed by N. E. Gilhus and coworkers. MMP-3 is capable of degrading a variety of proteins, including agrin, which plays a critical role in neuromuscular signaling by controlling acetylcholine receptor clustering. A pathogenic role of MMP-3 in other neurological disorders has been suggested but not proven. We have examined the levels of MMP-3 in 124 MG patients and compared them to 59 multiple sclerosis (MS) patients, 74 epilepsy patients, 33 acute stroke patients, and 90 healthy controls. 15.3% of the patients in the MG group were MMP-3-positive (defined as higher than cut-off value 48 ng/mL) with very high mean MMP-3 concentration (79.9 ng/mL), whereas the proportion of MMP-3 positive patients in the MS (3.4%), epilepsy (6.7%), stroke (0%), and the control group (4.4%) was significantly lower. Mean MMP-3 concentration in the total MG group (25.5 ng/mL) was significantly higher than in the MS (16.6 ng/mL) and stroke group (11.7 ng/mL), but did not differ significantly from the epilepsy (19.4 ng/mL) and the control group (23.4 ng/mL).

The Lambert-Eaton Myasthenic Syndrome (LEMS) is a rare disease with a well-characterized pathogenesis. It has many clinical and immunological similarities with MG, which are discussed by N. E. Gilhus. In 50% of the patients, LEMS is a paraneoplastic manifestation and is caused by a small cell lung carcinoma (SCLC). Both LEMS patients with SCLC and those without this tumour have in 85% of cases pathogenetic antibodies of very high LEMS specificity against voltage-gated calcium channels (VGCCs) in the cell membrane of the presynaptic motor nerve terminal. Better understanding of LEMS pathogenesis has lead to targeted symptomatic therapy aimed at the neuromuscular junction and to semispecific immunosuppression. For SCLC LEMS, tumour therapy is essential.

MG patients visiting outpatient clinics frequently complain of headache. There have been few reports on the relation between chronic headache and MG. N. Suzuki and co-workers have investigated whether MG symptoms affect the development or worsening of chronic headache. Among the 184 MG patients, tension-type headache was observed in 71 (38.6%) patients and 9 (4.9%) complained of migraine. Twenty-five (13.6%) complained that headache appeared or was exacerbated after the MG onset. The investigation into differences in the clinical characteristics of the MG patients showed that women tended to suffer from MG-associated headache more often than men. Logistic regression analyses revealed that female gender and mild ocular symptoms were independently predictive of headache associated with MG.

MG is characterized by reduced muscle endurance and is often accompanied by respiratory complications. Improvement of respiratory function is therefore an important objective in MG therapy. S. Hallebach et al. have examined long-term respiratory muscle endurance training in ten patients with mild-to-moderate MG. During the first month, they performed five training sessions per week. For the following 3 months, training frequency was reduced to five sessions per two weeks. Myasthenia score, lung function, and respiratory endurance were determined prior to training, after the first month and after 4 months. Myasthenia score improved from 0.71 ± 0.1 to 0.56 ± 0.1 (*P* = 0.007). Respiratory endurance time increased from 6.1 ± 0.8 to 20.3 ± 3.0 min (*P* < 0.001). The authors conclude that this maintenance program is feasible and is significantly beneficial for MG patients.

MG is of particular interest to anaesthetiologists because of the muscle groups affected, the pharmacology of the neuromuscular junction and interaction of both the disease and treatment with many anaesthetic drugs. Anaesthetists may encounter children with MG to facilitate treatment options or to institute mechanical ventilation in the face of a crisis. To complete the review on long-term treatment in MG, N. Bagshaw and W. Masters have reviewed the documentation pertaining to the pathophysiology and applied pharmacology of the disease and explore the relationship between these and the anaesthetic management.

*Johan A. Aarli* *Johan A. Aarli* *Nils Erik Gilhus* *Nils Erik Gilhus* *Robert P. Lisak* *Robert P. Lisak* *Renato Mantegazza* *Renato Mantegazza* *Shigeaki Suzuki* *Shigeaki Suzuki*

